# Pulse wave and vector flow Imaging for atherosclerotic disease progression in hypercholesterolemic swine

**DOI:** 10.1038/s41598-023-32358-1

**Published:** 2023-04-18

**Authors:** Paul Kemper, Grigorios M. Karageorgos, Daniella Fodera, Nicole Lee, Nirvedh Meshram, Rachel A. Weber, Pierre Nauleau, Nima Mobadersany, Nancy Kwon, Kristin Myers, Elisa E. Konofagou

**Affiliations:** 1 Department of Biomedical Engineering, Columbia University, New York, 10027 USA; 2 Department of Mechanical Engineering, Columbia University, New York, 10027 USA; 3 Department of Radiology, Columbia University, New York, 10027 USA

**Keywords:** Biomarkers, Diseases, Health care, Medical research, Engineering

## Abstract

Non-invasive monitoring of atherosclerosis remains challenging. Pulse Wave Imaging (PWI) is a non-invasive technique to measure the local stiffness at diastolic and end-systolic pressures and quantify the hemodynamics. The objective of this study is twofold, namely (1) to investigate the capability of (adaptive) PWI to assess progressive change in local stiffness and homogeneity of the carotid in a high-cholesterol swine model and (2) to assess the ability of PWI to monitor the change in hemodynamics and a corresponding change in stiffness. Nine (n=9) hypercholesterolemic swine were included in this study and followed for up to 9 months. A ligation in the left carotid was used to cause a hemodynamic disturbance. The carotids with detectable hemodynamic disturbance showed a reduction in wall shear stress immediately after ligation (2.12 ± 0.49 to 0.98 ± 0.47 Pa for 40–90% ligation (Group B) and 1.82 ± 0.25 to 0.49 ± 0.46 Pa for >90% ligation (Group C)). Histology revealed subsequent lesion formation after 8–9 months, and the type of lesion formation was dependent on the type of the induced ligation, with more complex plaques observed in the carotids with a more significant ligation (C: >90%). The compliance progression appears differed for groups B and C, with an increase in compliance to 2.09 ± 2.90×10^−10^ m^2^ Pa^−1^ for group C whereas the compliance of group B remained low at 8 months (0.95 ± 0.94×10^−10^ m^2^ Pa^−1^). In summary, PWI appeared capable of monitoring a change in wall shear stress and separating two distinct progression pathways resulting in distinct compliances.

## Introduction

Atherosclerosis, the formation and accumulation of lesions in the artery wall, is responsible for significant morbidity and mortality worldwide^1^. Furthermore, atherosclerosis is not only a problem in developed countries. Currently, over 80% of all cardiovascular deaths occur in low- and middle-income countries^2^. For a long time, the size of the atherosclerotic plaque and the degree of luminal narrowing were believed to be most predictive of ischemic cardiovascular disease risk^3,4^. Recent advancements in in vivo imaging of atherosclerosis have yielded critical new insights indicating that the fragility of an atherosclerotic plaque depends more on its composition than on lumenal constriction^5^. The stiffness or compliance of the plaque is related to its composition, with soft components being linked to more vulnerable plaque features and stiff components linked to more stable plaque components such as a thick fibrous cap^6,7^.

Additionally, there remains limited research regarding atherosclerosis initiation and progression. A better understanding of the onset of atherosclerosis and the ability to monitor progressive vascular damage leading to atherosclerotic lesions, could potentially help identify those on a path of future cardiovascular disease, such as complications from atherosclerosis. In this background, various flow-based markers, such as wall shear stress, are expected to play a significant role in the initiation of plaque lesions. As a result, while local stiffness is expected to be correlated with plaque and vascular composition, flow-based markers such as wall shear stress, which is an important driver in remodeling, are expected to dictate plaque progression and initiation^8–10^.

Studying atherosclerosis at the bifurcation of the carotid artery is crucial for various reasons. First, the bifurcation of the carotid artery is easily accessible for imaging. Second, atherosclerosis in the bifurcation of the carotid artery plays a significant role in the onset of ischemic stroke^11,12^. Thirdly, there is a strong association between the composition of atherosclerosis in the carotid bifurcation and plaque composition in the coronary arteries^13,14^, for which comprehensive imaging is difficult due to the small size of the arteries and cardiac motion^15^.

**Figure 1:**
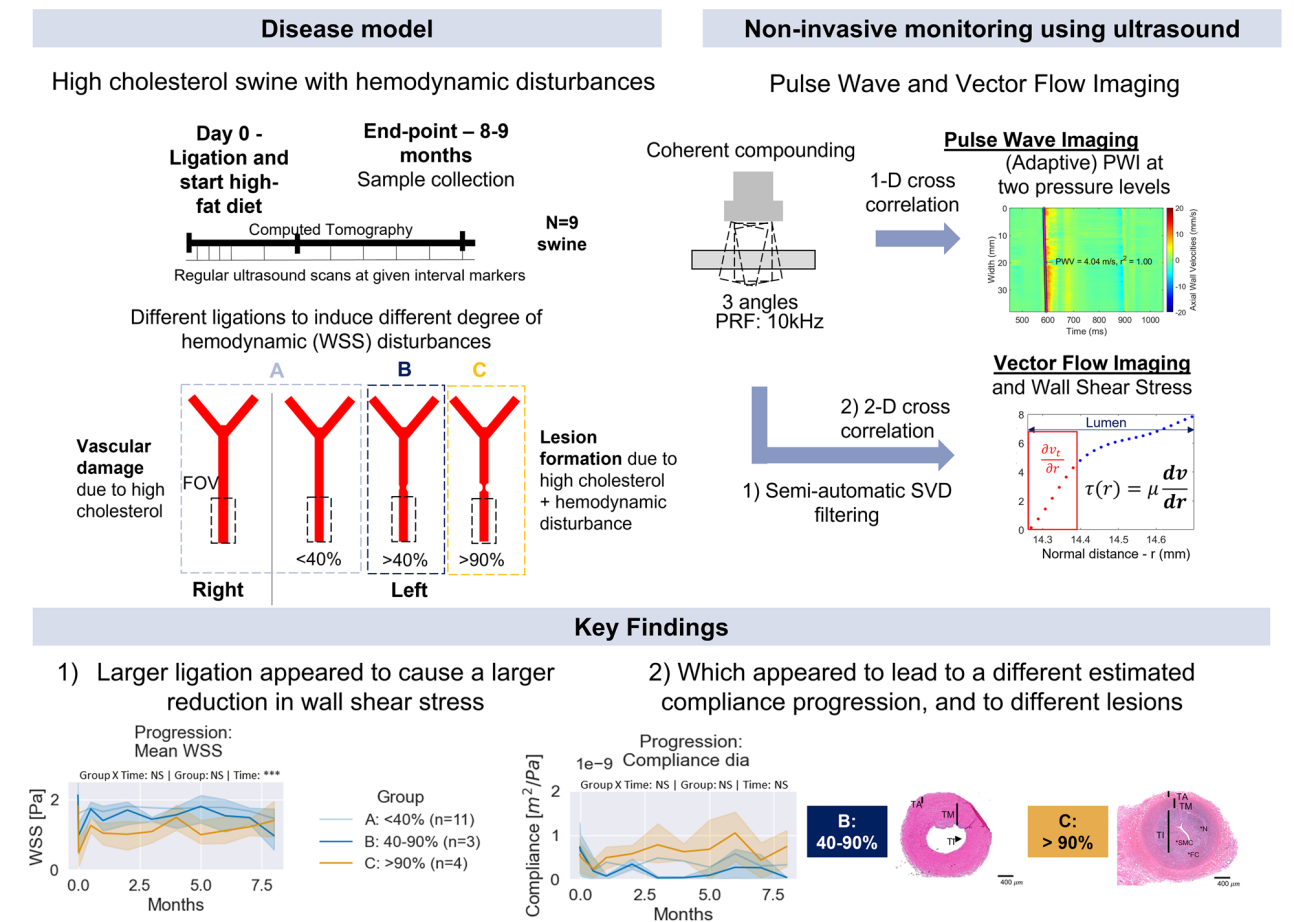
Central figure. A swine model with elevated cholesterol levels was used in this study to simulate the progression of atherosclerotic disease. Various degrees of hemodynamic disturbances were induced in order to accelerate the progression of atherosclerosis and to ensure that it is known where the plaques originate so that it can be monitored. The hemodynamic and stiffness markers were calculated using Pulse Wave and Vector Flow Imaging. The key finding was that a greater ligation appeared to reduce wall shear stress more drastically. This distinct observed hemodynamic disturbance seemed to have resulted in a distinct estimated progression of compliance and distinct lesions.

Various imaging modalities have been used to assess the local properties of the vasculature and plaque components^16–18^. Ultrasound is a cost-effective and non-invasive modality that is promising for assessing the stiffness of plaques using various techniques such as arterial wall strain imaging^19,20^, acoustic Radiation Force Imaging (ARFI)^21^, and Shear Wave Imaging (SWI)^21,22^. Ultrasound excels in temporal and spatial resolution compared to computed tomography and magnetic resonance imaging. Furthermore, ultrasound is currently the modality of choice to measure the degree of luminal narrowing, which is still often used as the sole marker for eligibility for treatment or surgery, making introducing additional techniques in clinical decision-making relatively straightforward. Finally, because of the cost-effective nature of ultrasound, it could also be used for screening purposes to identify early-stage asymptomatic atherosclerosis.

Pulse Wave Imaging (PWI) is a non-invasive technique to measure the local stiffness at diastolic or end-systolic pressure without the need for an external pressure value. This was achieved by tracking the propagation of pulse waves along the wall of the vessel at high spatial and temporal resolutions using high-framerate ultrasound (US)^23^. This pulse wave velocity is related to the compliance of the vessel as described by the Bramwell-Hill equation^24^. This technique has previously been validated in simulations and phantoms^25–27^, as well as in-vivo for for mice^28,29^, swine^30^, and humans^31^. Recently, PWI has been incorporated with vector flow imaging, providing a comprehensive framework to assess vascular stiffness and hemodynamics together^32–34^. While work has been done before that shows the ability of PWI to monitor plaque progression in mice, atherosclerosis in mice is different from that in humans.

Swine constitute a suitable model for atherosclerosis in humans^35^, and provide the opportunity to assess the ability of PWI to monitor the progression of early-stage atherosclerosis, as well as assess the stiffness of the plaque region. High-cholesterolomic swine, which mimic the disease of familial hypercholesterolemia, are of particular interest^36^. In humans, the long exposure to high cholesterol causes progressive damage to the vascular system and increases the risk of cardiovascular disease before the age of 60^37^. In swine, this leads to spontaneous plaque formation within 2 years, which can be further accelerated by artificially inducing flow disturbance^35^. In previous studies published by our lab, PWI was shown to be repeatable in swine during the progression of atherosclerosis in three swine^29^, However, due to the low sample size (n = 3), limited assessment could be made about the ability of PWI to monitor different plaque progressions. Additionally, the hemodynamic component was ignored. Recently, an adaptive wall shear stress algorithm was developed by our group, and feasibility was shown in phantoms, simulations, clinic and swine (n=6)^34^. Ligated arterial segments exposed to low wall shear stress developed moderate to severe stenosis (*p*<0.05). However, it was not possible yet to differentiate between different types of plaques between swine, and stiffness was not considered.

This study follows up on the pilot study and has two aims, namely: (1) to investigate the capability of adaptive PWI to assess progressive change in local stiffness of the carotid in a high-cholesterol swine model; and (2) to assess the ability of PWI to monitor change in wall shear stress and corresponding change in stiffness of the carotid over the course of the study. Nine hypercholesterolemic (n=9) swine are included in this study and followed for up to 9 months. A hemodynamic disturbance was achieved by inducing a ligation. The degree of ligation was classified using computed tomography and clinical ultrasound, after which the left and right carotids (n=18) were grouped into three groups, A: <40% ligation (n=11), B: 40–90% ligation (n=3) and C: >90% ligation (n=4). The hemodynamics were quantified using vector flow imaging and wall shear stress estimation, and the subsequent change in stiffness was monitored using adaptive PWI. The pulse wave velocity at end-diastolic, when the heart starts to contract, and at end-systole, when the valve close, were used to estimate the compliance at diastolic and end-systolic pressure. Finally, ex vivo comparison with histology (n=18) and nanoindentation (n=6) testing was performed to assess vascular damage and lesions (Fig. 1).

**Figure 2:**
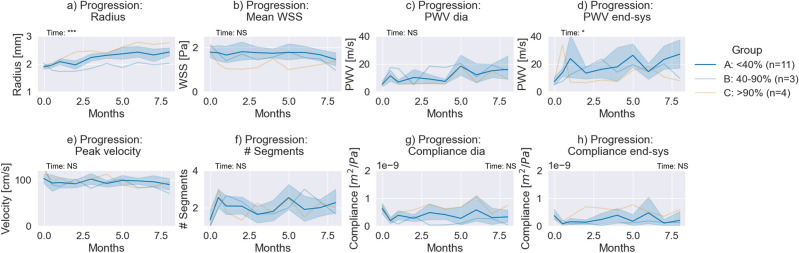
Progression of derived markers for the non-ligated carotids. The stiffness of the vessel, indicated by the compliance (**g**,** h**) and the PWV (**c**,** d**) increases over the course of the study, indicating vascular damage. (**a**) The radius of the carotid lumen. (**b**) The estimated temporal-averaged wall shear stress. **c**, The pulse wave velocity at diastolic (dia) pressure. (**d**) The pulse wave velocity at end-systole (end-sys). (**e**) The peak velocity. (**f**) The number of detected segments. (**g**) The compliance at diastolic (dia) pressure. (**h**) The compliance at end-systole (end-sys). The data are represented as the mean (bold) and the 95% confidence interval (transparent). One-way repeated measures ANOVA was conducted to quantify the significance (*<0.05, **<0.01 and ***< 0.001, with Holm-Sidak correction).

**Figure 3:**
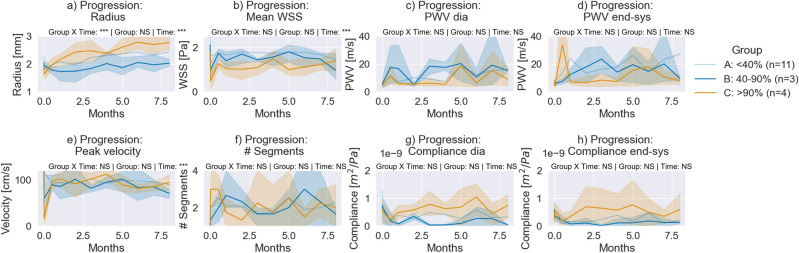
Progression of derived markers for the ligated carotids of groups B (blue) and C (orange). The stiffness of the less ligated group (40–90%) increases as indicated by the compliance (**g**,** h**) and the PWV (**c**,** d**), whereas the stiffness of the ligated group decreases. Furthermore, a distinct progression in the radius can be observed (**a**). (**a**) The radius of the carotid lumen. (**b**) The estimated temporal-averaged wall shear stress. (**c**) The pulse wave velocity at diastolic (dia) pressure. (**d**) The pulse wave velocity at end-systole (end-sys). (**e**) The peak velocity. (**f**) The number of detected segments. (**g**) The compliance at diastolic (dia) pressure. (**h**) The compliance at end-systole (end-sys). The data are represented as the mean (bold) and the 95% confidence interval (transparent). Two-way mixed ANOVA was conducted to quantify the significance (*<0.05, **<0.01 and ***< 0.001, with Holm-Sidak correction).

## Results

### In-vivo findings

#### Group A (<40% ligation)

Fig. 2 demonstrates the progression of estimated markers for the non-ligated carotids in group A (n=11 carotids). One-way repeated measures ANOVA was conducted to quantify the significance of the changes. In general, the stiffness of the vessel, as measured by compliance and PWV, increases throughout the study, indicating vascular damage caused by high cholesterol.

The pulse wave velocities at diastole ($p_{adjusted} = 0.046$) changed significantly over the course of the study. The pulse wave velocity at diastole increased after the start of the study (5.61 ± 4.38 ms^−1^ to 11.61 ± 7.06 ms^−1^, and further increased to 16.24 ± 15.9 ms^−1^ at 8 months. The pulse wave velocity at end-systole follows a similar pattern, and seems to increase from 7.51 ± 5.61 m s^−1^ to 14.03 ± 5.12 m s^−1^ in two weeks and then to 27.28 ± 18.29 ms^−1^ at 8 months post-op.

The compliance at diastole ($p_{adjusted} = 0.44$) and end-systole did not significantly change ($p_{adjusted} = 0.45$). The carotids showed constant peak flow velocity (102.6 ± 19.7 cm s^−1^ to 90.2 ± 20.81 cm s^−1^) and wall shear stress (1.76 ± 0.49 Pa to 1.46 ± 0.51 Pa) over the duration of the study. The number of detected segments appeared to change over the course of the study ($p_{adjusted} = 0.27$) within two weeks, and remained higher (1.5 ± 1.03 # at baseline and 2.00 ± 1.19 # at 8 months).

**Figure 4:**
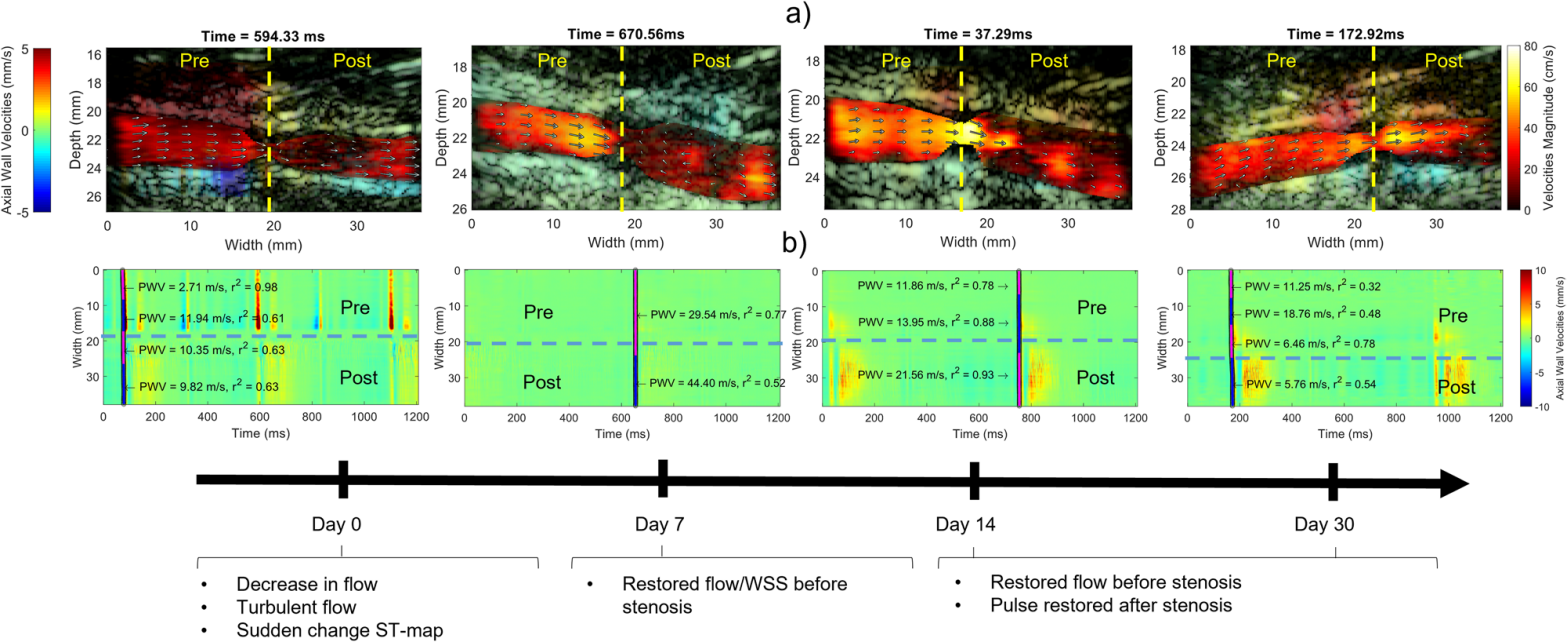
Qualitative example of early remodelling around the artificial ligation. (**a**) B-Mode of carotid around the artificial ligation with the wall velocity (left color bar) and flow velocity (right color bar) overlayed. Immediately after ligation the flow is reduced as indicating by a dark red color. Additionally, the pulse is obstructed, as seen by large wall velocities before the ligation and low wall velocities after the ligation. The flow has recovered in as little as 7 days, as indicated by a more yellow color indicating higher flow velocity. The pulse is not clearly visible anymore. After 14 days, the flow remains restored, and the pulse becomes more apparent again. (**b**) Spatio-temporal maps of the wall velocities. The y-axis corresponds to the position in the vessel, whereas the x-axis corresponds to the time. Similarly, as in (**a**) immediately after ligation, an obstruction in pulse propagation can be observed. The pulse is barely visible after 7 days, and becomes apparent again after 14 days. Following the ligation, the spatiotemporal map remains consistent.

#### Group B and C (40–90% and >90% ligation)

Fig. 3 demonstrates the progression of estimated markers for the ligated carotids in groups B (n=3 carotids) and C (n=4 carotids) of the acquisition located just before the induced ligation. A two-way mixed ANOVA was conducted to quantify the significance of the changes. The carotid with higher flow disturbance, as indicated by a large reduction in wall shear stress, revealed a decreasing radius of the lumen and a softening of the vessel, whereas the vessel with lower flow disturbance showed a more similar progression as the group B, with stiffening as indicated by the increase in pulse wave velocity and compliance. The peak flow velocity changed over the course of the study (time: $p_{adjusted} < 0.001$). The peak flow velocity decreased for group C (122.22 ± 36.63 cm s^−1^ to 15.61 ± 13.33 cm s^−1^) and group B (127.36 ± 34.75 cm s^−1^ to 59.56 ± 45.06 cm s^−1^) immediately after ligation. Subsequently, it returned to values close to the baseline. The average wall shear stress changed over the course of the study (time: $p_{adjusted} < 0.001$). The wall shear stress decreased immediately after ligation for group B (2.12 ± 0.49 Pa to 0.98 ± 0.47 Pa) and group C (1.82 ± 0.25 Pa to 0.49 ± 0.46 Pa). This distinction vanished after week two. The number of detected segments did not change significantly over the course of the study, but appeared to increase immediately after ligation for group C (1 # to 3 ± 1.4 #) and group B (1 # to 1.3 ± 0.58 #). Fig. 4 demonstrates this early remodelling process in a swine of group B. A quick recovery of flow can be observed. Immediately after ligation, a clear interruption of the pulse can be observed. As soon as six days later, the wall velocities both before and after the ligation had reduced, as had the flow and the wall shear stress. After 14 days, the pulse appeared after the ligation, and the spatiotemporal map around the ligation remained consistent after.

The pulse wave velocity at diastole or end-systole did not change significantly over the course of the study (time: $p_{adjusted} = 0.45$ and $p_{adjusted} = 0.43$). The progression of compliance at diastole was almost different for group B and group C (interaction: $p_{adjusted} = 0.09$), indicating a different compliance progression for group B and C. Initially, the compliance at diastole decreased from 5.79 × 10^−9^ m^2^ Pa^−1^ to 2.39 × 10^−10^ m^2^ Pa^−1^ for Group C 7.41 × 10^−9^ m^2^ Pa^−1^ to 2.38 × 10^−10^ m^2^ Pa^−1^. At the end-point, the compliance of group C did increase to 7.48 ± 5.02 × 10^−10^ m^2^ Pa^−1^ whereas the compliance of group B remained low at 8 months (0.53 ± 0.50 × 10^−10^ m^2^ Pa^−1^). A similar trend can be seen with the end-systolic compliance.

**Figure 5:**
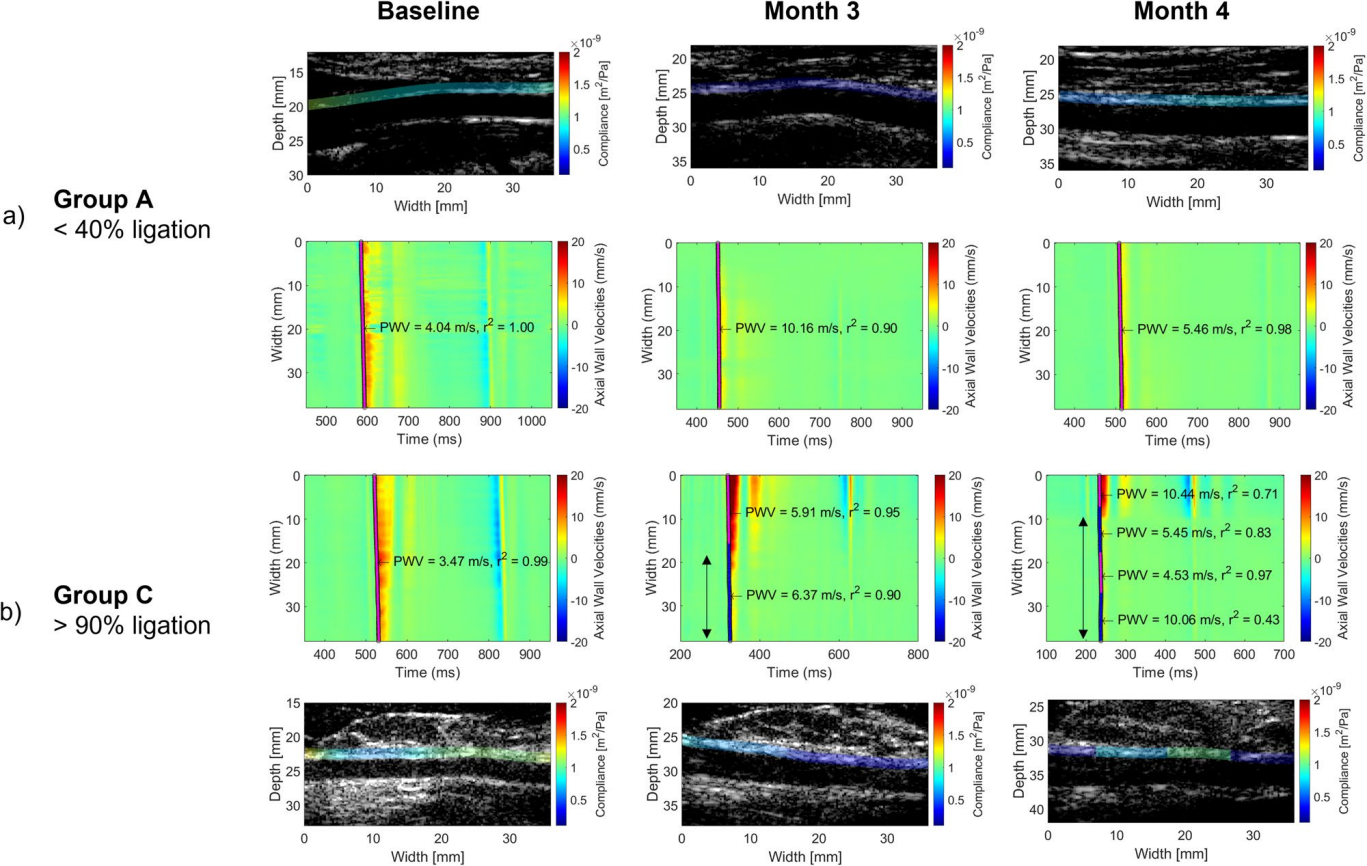
A qualitative example of remodeling over the course of the study and its impact on the spatiotemporal map for group A (**a**) and group C (**b**). The y-axis corresponds to the position in the vessel, whereas the x-axis corresponds to the time. (**a**) A homogenous reduction in wall motion can be observed, combined with an increase in PWV at months 3 and 4 compared to baseline. (**b**) An increased region of reduced wall motion can be observed in the same field of view, indicating a progressive change. At 3–4 months, multiple segments are detected, indicating inhomogeneous pulse wave propagation. A stiffer vessel can be observed after 3 months. After 4 months, the compliance values become more variable.

Fig. 5a demonstrates the initiation of plaque formation in an example of group A. At baseline, higher compliance, indicating a less stiff carotid, can be observed. Additionally, the pulse wave velocity was relatively homogeneous. At 3 months, lower displacement values can be observed over the entire width of the vessel. Furthermore, the pulse wave velocity has increased, indicating a stiffer vessel. At 4 months, a similar pattern can be observed, although with a pulse wave velocity above baseline and lower wall displacement velocities. Fig. 5b demonstrates the initiation of plaque formation in an example of group C. At baseline, higher compliance, indicating a less stiff carotid, can be observed. Additionally, the pulse wave velocity was relatively homogeneous, and the observed variability in compliance was due to a change in the measured radius. At 3 months, lower displacement values can be observed toward the ligation. At 4 months, this region increased while the field of view of the acquisitions remained the same. Additionally, variable pulse wave velocities and multiple segments can be observed.

**Figure 6:**
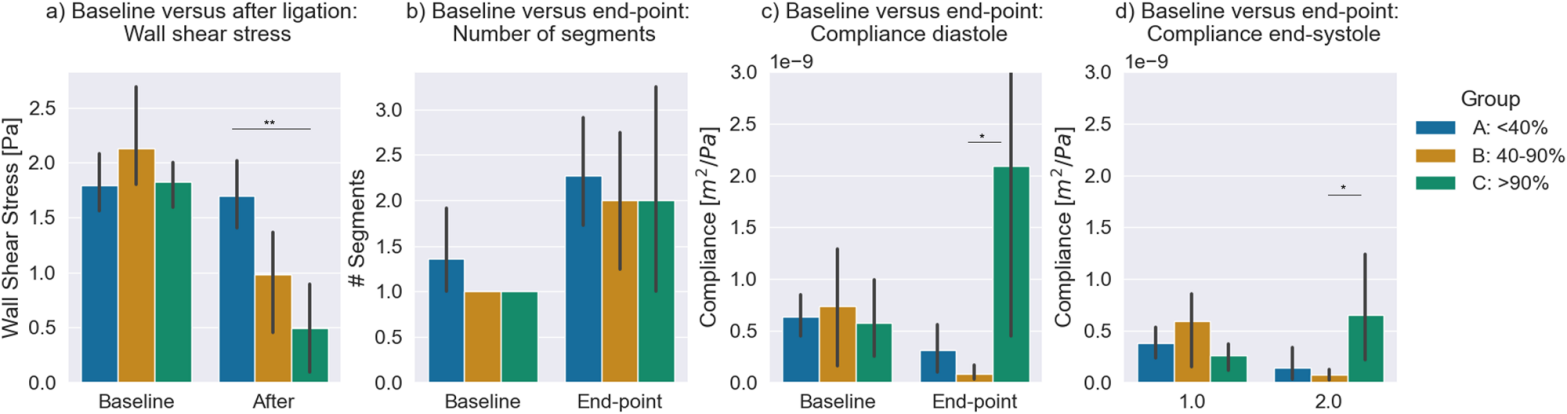
Quantitative comparisons at specific time-points. (**a**) The wall shear stress at baseline and first follow-up measurement (**b**) The number of detected segments at baseline and last measurement (**c**) The compliance at diastole at baseline and last measurement (**d**) the compliance at end-systole at baseline and last measurement. The 95% confidence interval is indicated by the black bar. Unpaired* t* test was performed between groups to quantify significance. (*<0.05, **<0.01 and ***< 0.001).

**Figure 7:**
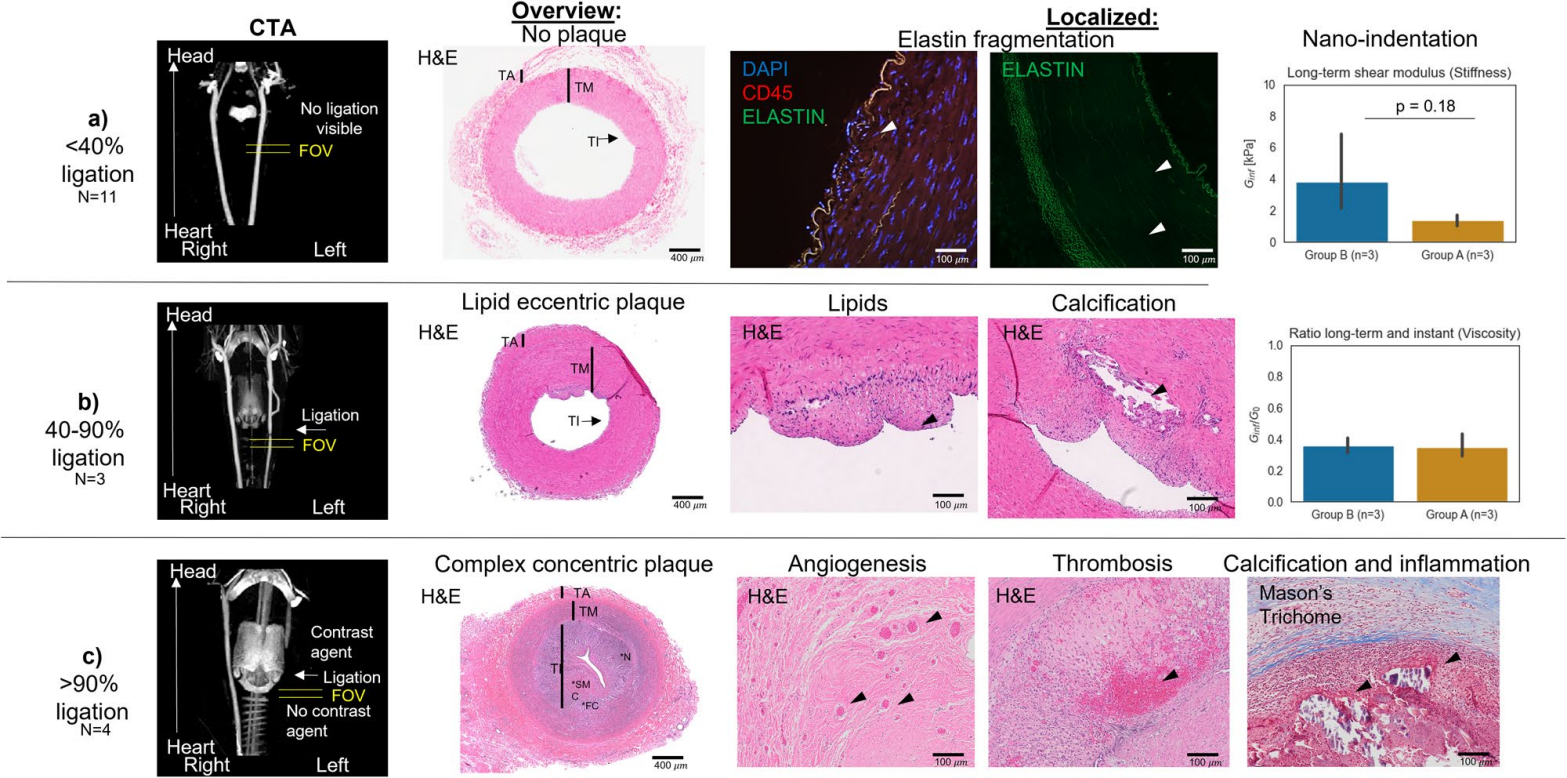
Qualitative example of histology. Significant plaque development can be observed in the >90% group, whereas plaque with less advanced features can be observed in the 40–90% group. While no plaque features were observed in group A, elastin fragmentation was observed as indicated by an interruption of the elastin fiber. Nanoindentation results showed a non-significant (*p*=0.18) increased long-term relaxation shear modulus $G_{inf}$ indicating that group B appears to have more stiff components than group A. No difference in viscosity was observed between the two groups. Abbreviations: H & E: hematoxylin and eosin. CTA: computed tomography angiography. TA: tunica adventitia. TM: tunica media. TI: tunica intima. FC: foam cells C: calcification. N: necrotic SM: smooth muscle cells.

### End-point and ex vivo comparison

Quantitative comparisons at specific time-points were performed, as demonstrated in Fig. 6a,b,c. In general, the observed differences observed in groups B and C using the non-invasive biomarkers agreed with ex-vivo comparison. To determine significance, an unpaired* t* test was used between groups. At baseline, no significant difference in wall shear stress was observed between the groups. In the first measurement after ligation a significant lower wall shear stress was observed between the <40% and >90% ligation (1.68 ± 0.53 Pa and 0.49 ± 0.46 Pa ) Fig. 6a. For group A, B, and C no significant differences were observed in the number of detected segments at baseline or end-point Fig. 6b. No significant differences were observed in the diastolic compliance at baseline, while significant difference between group B and C were observed for the end-point measurements (9.51 ± 9.43 × 10^−11^ m^2^ Pa^−1^ and 2.09 ± 2.90 × 10^−10^ m^2^ Pa^−1^). Similarly, for the end-systolic compliance, no significant differences were noted for the end-systolic compliance at baseline, while significant differences between groups B and C were observed for the end-point measurements (7.51 ± 7.42 × 10^−11^ m^2^ Pa^−1^ and 6.56 ± 6.04 × 10^−10^ m^2^ Pa^−1^).

Fig. 7 reveals the ex-vivo quantification of the carotids of the three groups. Group C, with the most compliant carotids, showed complex plaque features such as angiogenesis, thrombosis, minor calcification, and inflammation. Group B showed some minor lipid depositions and some features of a complex plaque, but less than group C. Both groups A and B showed signs of elastin fragmentation. Additionally, nanoindentation indicated a non-significant (*p*=0.18, paired* t* test) increase in mean long-term shear moduli for group B (n=3) compared with group A (n=3). The mean long-term shear moduli for the three carotids of Group B is 3.80 ± 2.62 kPa and the mean long-term shear moduli for the three carotids of Group A is 1.36 ± 0.33 kPa. The ratio of the long-term shear moduli with the instant shear moduli provides a measure for viscosity. Both group A and B have a similar ratio with 0.35 ± 0.07 [−] for group A and 0.36 ± 0.04 [−] for group B.

## Discussion

Pulse Wave Imaging in combination with vector flow imaging is a promising tool for monitoring atherosclerosis initiation and progression. However, it remains unclear whether the derived wall shear stress and compliance markers are sufficient to assess different types of plaques. This study looked at two things: (1) the ability of compliance, as measured by tracking the local pulse wave velocity, to monitor progression of local stiffness and homogeneity of the carotid in a high-cholesterol swine model, and (2) the ability of PWI to monitor change in wall shear stress and corresponding change in carotid stiffness.

The study presented herein found that the carotids without artificially induced flow disturbance showed signs of progressive damage. The pulse wave velocity at the end-point of 8–9 months (16.24 ± 15.9 ms^−1^) is significantly higher compared to baseline (5.61 ± 4.38 ms^−1^) (Fig. 2). This is in agreement with the Rotterdam study^38^, which found increased aortic pulse wave velocity even in mild-atherosclerotic cases. It is also in agreement with previous work in mice that showed elevated pulse wave velocity in the early-stages of atherosclerosis in mice^28^. Elastin fragmentation was observed in histology, as seen in Fig. 5 indicating vascular damage. Elastin fragmentation is related to atherosclerosis via various pathways^39^. Additionally, elastin fragmentation is observed in heart valve leaflets in Rapacs familial hypercholesterolemia swine^40^, indicating that this might be a systemic consequence of familial hypercholesterolemia in swine. Elastin fragmentation potentially explains the increased pulse wave velocity due to more load on the stiffer collagen fibers. Reduction of elastin has shown to cause a decrease in compliance^41,42^. Increased bearing of pressure by the collagen fibers during the cardiac cycle are also expected to be responsible for observed non-linear material properties, and why the pulse wave velocity at diastole is lower than the pulse wave velocity at end-systole at baseline (5.61 ± 4.38 ms^−1^ versus 7.51 ± 5.61 ms^−1^) and 8 months (16.24 ± 15.9 ms^−1^ versus 27.28 ± 18.29 ms^−1^). The non-invasively obtained number of detected linear pulse wave propagation segments is correlated to inhomogeniety, since any homogeneity in stiffness and/or geometry causes increased non-linearity in pulse propagation. While not significantly different, they appear to change and increase over the course of the study (Fig. 6). Whereas the pulse wave velocity is significantly different, this is not the case for the compliance. This does not necessarily mean that the material properties do not change over the course of the study. First, the compliance is a marker which incorporates both the radius and the pulse wave velocity, both having their measurement biases. These biases propagate in the estimation of compliance. This was previously shown in the feasibility study of pulse wave imaging in swine, in which the intra-user variability of the compliance can be as high as 20%^30^. Second, the compliance is not the same as the material properties, meaning a bigger carotid with the same vascular composition has a higher compliance than a smaller carotid. Since the radius increases significantly over the course of the study, this leads to an apparent softening of the vessel. For conversion to an actual material property, such as the Young’s modulus, accurate wall thickness is required, which was not obtained in this study. Furthermore, additional assumptions, such as thin-wall, have to be made when using, for example, the Moens-Korteweg equation^43^. This might not be valid in atherosclerotic vessels, which tend to have a larger wall thickness.

Second, the study revealed that the carotids with flow disturbances showed signs of lesion formation. Two different types of lesion formation were observed for the group with most significant flow disturbance and the one with the least severe flow disturbance. This finding is in agreement with a previous study, in which wall shear stress was shown to influence remodeling^44,45^. For group C (>90% ligation), more compliant vessels were observed. Interestingly, less compliant vessels were observed for the vessels with less severe flow disturbance (group B). The observation that the lipid plaque component results in less compliant arteries is consistent with prior studies in humans^28^ and, to a lesser extent, mice^25^. Despite the small sample size used (n=3 and n=4), the different progression was statistically significant as indicated by the repeated measures mixed ANOVA (interaction: $p_{adjusted} = 0.09$). It seems that the type of flow disturbance initiates two different paths of plaque formation, namely, (1) constant radius and stiffening, and (2) dilation and softening. The complex plaque formation was confirmed with histology (Fig. 7). Lipid-plaques were observed in group B, with some more complex features such as calcification before the ligation. Nanoindentation revealed increased mean long-term shear moduli for the case with flow disturbances (group B). This indicates that stiffer components were present in the regions tested, which is in agreement with the findings of histology, in which some signs of calcification were observed in the carotids of group B. Thus, suggesting while that just familial hypercholesterolemia induce signs of vascular damage, flow disturbance and/or low wall shear stress induce remodelling that leads to more complex plaque formation.

Although the methodology used was sufficient to observe different progressions in this study, there remain various limitations. The derived compliance value requires an estimation for the lumenal area, and due to the 2-D acquisitions, some assumptions must be made. The assumption of a circular lumen, in particular, may be violated in carotids with plaques. Additionally, reflections can occur, which might impact the compliance measures. In this study, this is expected to play a more significant role for the ligated carotids. The pulse is visibly disturbed, as indicated by an increase in detected segments immediately after ligation. While this shows the sensitivity of the adaptive method to detect inhomogeneities, even outside the field-of-view, it could bias the obtained compliance values of the ligated carotids. Furthermore, compliance is a composite measure of the properties of the vascular wall and plaque. Future work in separating the properties of the plaque and the wall could be done by, for example, by acquiring 3-D or cross-sectional data and measuring the local displacement values.

Other biomarkers of relevance, such as turbulence, can be derived from vector flow imaging^46,47^. This turbulence will be most noticeable after the ligation and will cause substantial out-of-view flow. A significant downside of any wall shear stress estimation is its sensitivity since it is based on the derivative of the flow profile at the wall. Additionally, especially in complex flow patterns, the assumption that all components of the flow vector are contained within a 2-D slice is likely not met. To avoid this problem, only the segment preceding the ligation is examined to track the evolution of the various markers. This is not a significant issue for the wall shear stress values used in this study. The geometry of the pre-ligation segment is relatively symmetrical, and no turbulence was observed in the pre-ligated segment. Therefore, the flow components still remain mostly within the 2-D field-of-view. Additionally, in this animal model, the observed reduction of the wall shear stress is mostly due to a reduced flow velocity. The main benefit of the adaptive wall shear stress technique utilized is that it is more robust against erroneous wall segmentation, which improves the robustness of the wall shear stress estimates. In future work, the adaptive WSS technique could be extended to 3-D flow, which would solve the 2-D assumption. Further improvements in the vector flow imaging methodology will subsequently improve the wall shear stress estimation as well. Whereas the lesions in this study appeared to be relatively diffuse, lesions in clinic are often more focal and occur at sites of significant reflection, such as the bifurcation. This complicates pulse wave tracking due to the changing pulse wave shape. To address this, current research is focused on the inverse problems that can resolve reflections and signal processing techniques that can suppress the impact of a reflection on wave-tracking based pulse wave velocity.

The observed variability in the induced degree of stenosis was not planned for and was due to experimental variability. Because of the observed differences in progression, particularly in the radius of the lumen, it was necessary to analyze them as separate groups. However, this came at the expense of the study’s statistical power. The study’s low power must be considered when interpreting the findings, and the study should be viewed as a pilot study. Furthermore, because the groupings were not necessarily planned, no random allocation was carried out. This could imply that some of the differences are explained by factors that have not been considered. The authors believe this is unlikely because the groupings continue to span multiple batches (i.e., Group A includes swine from all three batches spanning three years, and Group B/C includes swine from at least two batches) and the findings appear to be consistent.

The main clinical significance of this study is that the discovery that PWI can monitor plaque progression may be relevant in a patient who has had routine clinic follow-up tests. Ultrasound is frequently used in the clinic to detect plaque changes. Changes in plaque appearance, for example, are indicative of plaque risk^48^. Identifying a considerable change in pulse wave velocity and/or stiffness could be symptomatic of a change in plaque that would otherwise be missed if only the degree of stenosis was considered. The fact that PWI can monitor alterations in this controlled setting suggests that this may be the case in humans as well, giving an easy and objective approach for the assessment of changes in plaque composition beyond the anatomical stenosis, and aiding clinicians in determining if the patients need to undergo intervention.

In summary, the ability of PWI to monitor changes in vascular stiffness in a high-cholesterol swine model and the ability of PWI to monitor changes in hemodynamics and the resulting stiffness progression were assessed. PWI was able to monitor changes in vascular stiffness over time in a high-cholesterol swine model. Subsequently, it was able to monitor changes in wall shear stress and separate two type of vascular remodelling resulting in distinct compliances. This indicates that PWI can be used to monitor plaque progression, and that flow-based markers such as wall shear stress are measurable non-invasively in the carotid in this disease model, and potentially provide complementary information for monitoring by providing some information about the future state of the vessel. Ongoing studies focus at improving robustness using more complex model-based approaches and taking into account the 3-D geometry of the lesions.

## Methods

### Animal study

The procedures performed in this study were approved by the Institutional Animal Care and Use Committee of Columbia University (protocol AC-AAAU6460) and were carried out in accordance with relevant guidelines and regulations. Additionally, the study was in compliance with the ARRIVE guidelines. Nine (6 males, 3 females) 3-months old Wisconsin Mini Swine-Familial Hypercholesterolemic (WMS-FHTM) were acquired from the University of Wisconsin Swine Research Farm (Madison, WI, USA). Nine (n=9) was expected to be the minimal amount of swine needed to observe any significant differences in the compliance of different type of plaques based on the initial pilot study^29^. The weight of the swine was 17 ± 3 kg and the age was 3.5 ± 0.5 months at the beginning of the study. The animals were fed a high-fat diet (15% lard, 1.2% cholesterol) (UW Swine Research Farm, Madison, WI) and their left common carotids were partially ligated to induce hemodynamic instability. The experiments were performed in three batches of three (n=3) swine over 3-years. Before the ligation procedure, intravenous propofol was administered to the animals to induce anesthesia. The animals were then intubated and maintained under 1–2% isoflurane anesthesia throughout the procedure. To gain access to and dissect the left common carotid, a 5 cm incision was made in the middle portion of the neck. A 1.7 mm spacer (a 5F feeding tube/urethral catheter) was placed along the artery before tying it off with 5-0 Prolene (Ethicon, Cornelia, GA, USA). The spacer was then removed, resulting in carotid stenosis. The neck wound was then surgically sutured layer by layer, and the location of the ligation was externally indicated by a different suture or tattoo^29^. For several swine (n=3) telemetry EasyTEL (Emka Tech, Paris, France) device was implemented in the femoral artery to investigate whether any significant changes in pressure would occur during the study. The pressure remained constant during the course of the study (diastole: 82.66 ± 10.66 mmHg at begin to 89.33 ± 11.15 mmHg at 4-months, systole: 131.88 ± 4.43 mmHg at begin to 124.66 ± 18.71 mmHg at 4-months (n=3)).

**Figure 8:**
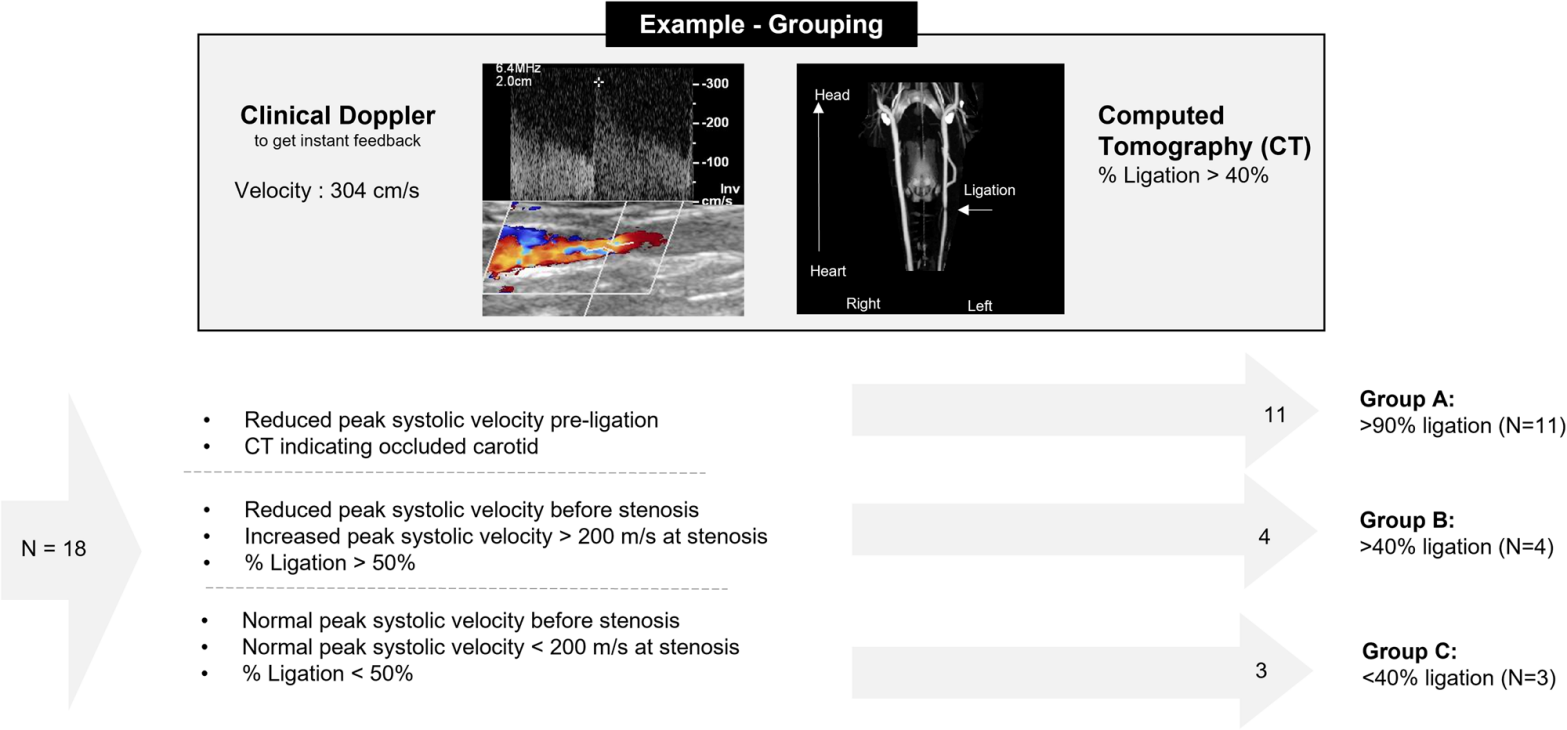
Qualitative example of grouping. The carotids in which no ligation was noticeable on computed tomography (CT) and/or clinical ultrasound were grouped into group A. When a narrowing was noticeable on computed tomography (and/or increased peak systolic velocity was measured at the ligation it was categorized as B. When almost complete ligation as observed on computed tomography and/or ultrasound it was categorized as group C.

Grouping was not done at random, but based on the variation in outcome of the ligation procedure. The degree of narrowing due to the ligation was assessed after ligation using clinical ultrasound and computed tomography angiography. A clinical ultrasound system (Epic CVx, Philips, Netherlands) with a hockey stick transducer (L15-7io, Philips, Netherlands) was used to assess local peak systolic flow velocity since it provides instant feedback and optimal alignment with the ligation is required. Computed tomography angiography was used to obtain document the assess the degree of narrowing as soon as a week and up to a month after induced ligation. The carotid arteries (n=18) of the nine (n=9) swine were divided into three groups based on the extent of luminal narrowing. Group A (n=11) consists of carotids without observable luminal narrowing after ligation (n=2), or carotids in which ligation was not performed (n=9). Group B (n=3) consists of carotids that showed signs of increased peak flow velocity (>200 ms^−1^) at site of ligation and/or observable narrowing on computed tomography angiography (>50%). Group C (n=4) consists of carotids with near complete occlusion as indicated by computed tomography angiography (Fig. 8).

**Figure 9:**
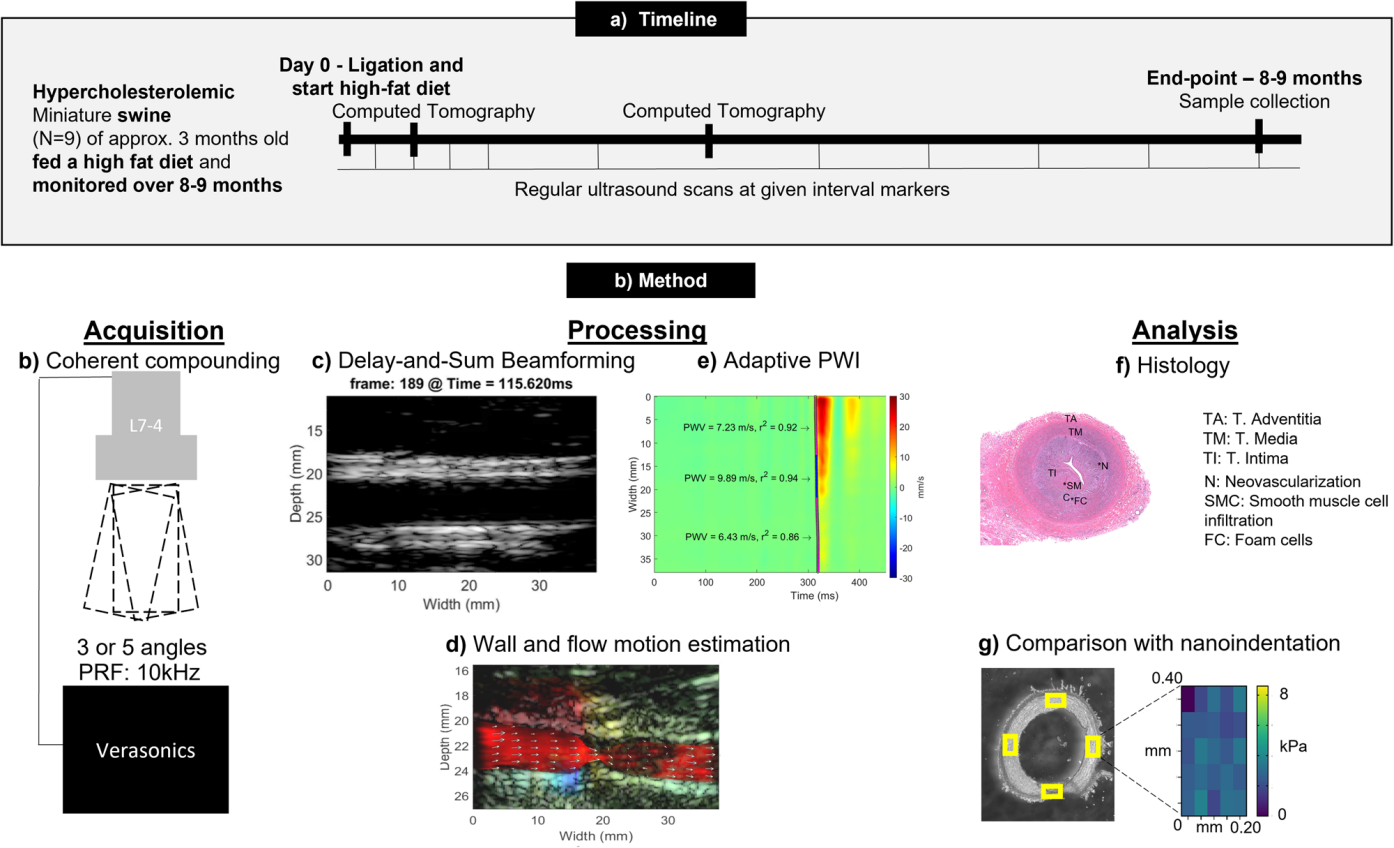
Study setup. (**a**) Timeline of the study. (** b**–**g**) Methodology of the study. (**b**) Coherent compounding is performed followed by (**c**) delay-and-sum beamforming. Subsequently, (**d**) Wall and motion estimation is performed followed by adaptive PWI (**e**) to assess hemodynamics and stiffness. Finally, (**f**) ex-vivo comparison is performed by histology and (**g**) nanoindentation.

The common carotids of the animals were imaged immediately prior and following the surgery (Fig. 9). The measurements were repeated two weeks after surgery and then once a month for 8 months. For three (n=3) swine additional measurements were made at 7 and 21 days. The animals were fed an atherogenic diet for the whole duration of the experiment. One of the animals expired at 8 months. The veterinary staff of the Institute of Comparative Medicine performed a necropsy and determined that the cause of death was a stroke due to a ruptured plaque^29^.

In the analysis of the progression of the markers, only the overlapping period (i.e. 8 months) were included, hence for eight (n=8) swine the last scan at 9-months is excluded. For a similar reason, the scans at 7 and 21 days were excluded in the progression comparison to ensure matching time-points. Additionally, only the region before the ligation was included for monitoring of biomarkers due to significant turbulence after the ligation. To ensure a fair comparison, the ex vivo comparison also focused on plaques that occurred before the ligation. The investigators were not blinded during scanning due to the difference in appearance of each swine, however, the ultrasound-data was processed in a randomized manner without knowledge to which swine the data belonged. During the necropsy, the common carotids were extracted. The swine were euthanized with an intravenous bolus of euthanasia solution (100 mg kg^−1^). Following euthanasia, an incision was made in the neck of the animals, and the common carotids were extracted, taking care to include the entire region imaged in the ultrasound acquisitions^29^. For end-point comparison, the last scan before extraction was used, thus at 9-months (n=8 swine) and at 8-months (n=1 swine) for the swine that died prematurely.

### High framerate US acquisitions

High frame rate (3.3kHz) ultrasound channel data were acquired using a 128-element L7-4 linear array (Microscan, Toronto, Ontario, Canada) operating at a central frequency (Fc) of 5 MHz and a Vantage 256 research ultrasound system (Verasonics, Seattle, Washington, United States) with a pulse repetition frequency of 10 kHz. Compounding with three plane wave angles (−10°, 0°, 10°) was used to ensure sufficient image quality and a high frame rate (3.3 kHz). It was employed with a pulse length of two cycles. This acquisition sequence was chosen because it has been demonstrated to meet the requirements for temporal resolution and imaging quality for PWI processing^25^, while the magnitude of the angles was such that flow data could be obtained with sufficient quality^32^. The acquisitions were performed under anesthesia with the ultrasound positioned on the neck of the swine. Beamforming was applied to the acquired channel data to produce full radio-frequency frame sequences. The beamforming employed in this study was a parallel pixel-wise implementation of the delay-and-sum (DAS) method^25^.

### Diastolic and end-systolic stiffness

The axial (parallel to the ultrasound beam or vertical dimension on the images) wall velocities were estimated from the compounded RF-frames. In this study, a sub-sample GPU-based 1-D normalized cross-correlation algorithm was used to obtain displacement values^49^. The obtained displacement values were filtered with a 3 × 3 × 3 samples median kernel for the 3-D matrix followed by first-order Savitzky-Golay filtering (4x4 samples) for each 2-D time-frame separately. The carotid wall was segmented manually at the wall-lumen interface to obtain the axial wall velocities of each wall at each lateral position along the imaged arterial segment. The posterior velocity was subtracted from the anterior velocity to eliminate possible rigid motion. This yields a spatiotemporal map of the axial distention rate, which has shown to provide more robust pulse wave tracking^33,50^. Subsequently, differentiation in the time domain was performed using a first-order Savitzky-Golay kernel (10 × 16 samples) to obtain the temporal waveform of the axial acceleration at each lateral position. The measured spatiotemporal map consisting of the incremental displacements of the carotid wall was used to derive a compliance value. This was achieved by obtaining two wave feature markers, namely a wave feature at diastolic pressure, and a wave feature at end-systolic pressure. Linear regression was performed to estimate the pulse wave velocity (PWV) corresponding to these different waves. To identify and quantify spatial variations in arterial wall properties across the imaged vessel adaptive PWI was utilized, which uses a graph-modelling framework in order to determine arterial segments where the pulse propagation is most homogeneous^31^. The number of detected segments are therefore related to how homogeneous the pulse propagation is, and expected to be correlated to vascular homogeneity. Finally, Bramwell-Hill equation is used to estimate the compliance $\left( {\frac{{dA}}{{dP}} = k_{p} } \right)$ corresponding to these two different pressure levels^24,51^.

1 $$\begin{aligned} PWV=\sqrt{\frac{A}{\rho k_{p}}}. \end{aligned}$$

where *PWV* is the estimated pulse wave velocity at diastolic pressure or end-systolic pressure, *A* is the luminal area at either diastole or end-systole and $\rho$ the wall viscosity.

### Flow and wall shear stress

Utilizing the same acquisition as acquired for PWI, the flow was estimated by tracking the 2-D motion of blood scatterers using speckle tracking^32^. The compounded radiofrequency signals were filtered using a singular value decomposition approach aiming to eliminate the slow motion of the arterial wall^52^. The threshold was determined in a semi-automatic manner utilizing the spatial similarity matrix. This approach was semi-automatic since sometimes manual post-correction was necessary. A 1-D normalized cross correlation kernel was subsequently applied on consecutive filtered RF frames in a 2-D search, using a kernel overlap of 1 axial sample (0.01848 mm), in order to estimate the inter-frame displacements of the blood scatterers. The size of the kernel was set at 80 (1.4784 mm). The resulting inter-frame displacements were then normalized by the frame rate in order to obtain the axial and lateral (flow velocity components. The resulting flow velocity components were averaged in temporal ensembles of 40 frames with 90% overlap. Finally, the estimates were spatially filtered using a 2-D median kernel of size 7 × 7. From the estimated 2−D+t flow velocity vector data *v* (*r*, *x*, *t*), the wall shear stress can be estimated:

2 $$\begin{aligned} \tau =\mu \frac{\partial v}{\partial r} |_{r=0}. \end{aligned}$$

Where μ is the fluid viscosity, *r* is a position in radius of the lumen with $r=0$ the lumen-wall boundary, *v*(*r*, *x*, *t*) is the flow vectors over time *t* at length *x* of the vessel, and *r*(*x*, *t*) the radius of the wall. In practice, the derivative term $\frac{dv}{dr}$ at $r=0$ is difficult to calculate exactly due to its sensitivity to inaccuracies in estimated flow data. Therefore the estimated vector flow data was used to estimate the wall shear stress using an adaptive approach^33^. Here, $\frac{\partial v}{\partial r}$ is approximated with $\frac{v(r) - v(r_0)}{r-r_0}$. Here $r_0$ is the location of the wall and *r* some position in the lumen. In the adaptive approach utilized, *r* and $r_0$ are not kept constant but optimized for each wall (both upper and lower) at each longitudinal position. This optimization problem is solved using* k*-means clustering based on several criteria. Specifically, the number of clusters, the shear rate, and the distance to the manually segmented wall. This criterion penalizes clusters that are not close to the wall, as well as clusters with low shear rates that may be the result of non-idealized wall clutter filtering and wall segmentation.

### Histology

After the swine were euthanized, the right and left carotid arteries was removed and placed in 1x PBS. A total length of approximately 200 mm was cut; 50–100 mm both before and after the point of ligation, washed in PBS then further sectioned in 1 mm segments which were further split in half to be split for histology and nanoindentation. Samples for nanoindentation were flash frozen then sectioned at 60 μm and collected onto microscope slides and kept at −80°C until ready for use. The other half of the segmented artery was fixed in 10% formalin for 24 h for histology. Samples underwent a dehydration series with ethanol to be embedded in paraffin then sectioned at 5 μm and collected onto a slide with a temperature-controlled water bath, then stained with hematoxylin and eosin (H & E) and Masson’s trichrome. Fluorescent microscopy was used, specifically DAPI to visualize nuclei and Cluster of differentiation (CD) 45 as a marker for inflammation. Additionally, auto-fluorescence of the tissue was exploited to visualize elastin.

### Nanoidentation

Spherical-nanoindentation (Piuma, Optics11 Life, Amsterdam, Netherlands) with a probe radius of 56 μm and a cantilever stiffness of 0.46 Nm^−1^ was utilized to determine the stiffness of the carotid arteries. Flash frozen tissue sections were thawed at room temperature and submerged in OptiFree Contact Solution (Alcon, Forth Worth, TX, USA) for testing at room temperature. Four distinct rectangular regions, sized 200 μm × 400 μm, were evenly distributed around the arterial wall tangential to the longitudinal fibers, yielding approximately 100 data points per tissue section. A fixed indentation depth of 5 μm, corresponding to 6% strain, was held for 20 s, yielding a load relaxation curve approaching equilibrium. Loading and unloading periods were each held constant at 2.5 s. The load relaxation curve was fitted with a generalized Maxwell model to determine the instantaneous and long time elastic moduli (Fig. 9g). The instant-term shear moduli for all locations were then grouped together for each carotid by averaging all data-points. The carotids where then grouped according to the degree of ligation as shown in Fig. 8. Three carotids (n=3) were grouped as group A, and three (n=3) were grouped as group B.

### Statistics

Python 3.9 with the Pingouin Toolbox was used for the statistical analysis^53^. The following ultrasound-based outcome variables were assessed: radius, wall shear stress, pulse wave velocity at diastole and end-systole, number of detected segments, compliance at diastole and systole, and peak blood flow velocity. Either one-way repeated measures or a two-way mixed ANOVA was used to quantify the progression of the different groups. The Holm-Sidak correction was performed to adjust the* P* values to control the familywise error rate because multiple parameters were investigated.

The first question was whether PWI could predict the progressive vascular damage caused by the high-fat diet. To test this, repeated measures ANOVA was done on the non-ligated carotids to see if any of the indicators changed significantly over the trial. The result of the one-way repeated measurements indicates whether one of the successive timepoints differs significantly from the others. Following that, we wanted to see if PWI could distinguish between two types of plaque advancement. To examine this, a repeated measures mixed ANOVA was used to determine whether groups B and C vary at any timepoint. The interaction (Group X Time) determines whether there is a timepoint for which the two groups are statistically different, (Group) indicates whether the two groups are different regardless of the time and the time, whether the timepoints are different regardless of the group. An unpaired* t* test was used to compare the end-points of interest of the three groups, and the Holm-Sidak correction was used to account for multiple comparisons (n=3). The time-point of interest for wall shear stress was selected immediately upon ligation, whereas the time-point of interest for the number of segments and compliance was the last measurement recorded for each swine, corresponding to just before the histological sample was extracted.

In all the statistical tests, the null hypothesis was rejected at the 0.05 level. Since the nanoindentation measurements were dependent (i.e., left-right carotid in the same swine), a paired* t* test was performed to quantify the significance of the differences between these two groups. The results are presented as the mean ± standard deviation, and the provided confidence interval in figures represents the 95% confidence interval of the mean.

## Data Availability

All data associated with this study are present in the paper. All data are available upon request from the corresponding author (E.K., ek2191@columbia.edu).
